# Pressurized intraperitoneal aerosol chemotherapy: a review of the introduction of a new surgical technology using the IDEAL framework

**DOI:** 10.1002/bjs5.50257

**Published:** 2020-01-19

**Authors:** S. J. Tate, J. Torkington

**Affiliations:** ^1^ Department of General Surgery University Hospital of Wales Cardiff UK; ^2^ Division of Cancer and Genetics Cardiff University School of Medicine Cardiff UK

## Abstract

**Background:**

The IDEAL (Idea, Development, Evaluation, Assessment, Long‐term study) framework is a scheme of investigation for innovative surgical therapeutic interventions. Pressurized intraperitoneal aerosol chemotherapy (PIPAC) is a procedure based on laparoscopy to deliver intraperitoneal chemotherapy for peritoneal metastases, introduced in 2011. The aim of this article was to review literature on PIPAC and assess whether development of the technique has followed the IDEAL framework.

**Methods:**

A search of MEDLINE and Embase was carried out to identify scientific reports on PIPAC published between January 2000 and February 2019. The studies were categorized according to the IDEAL stages.

**Results:**

Eighty‐six original research papers on PIPAC were identified. There were 23 stage 0, 18 stage 1, 25 stage 2a and six stage 2b studies. Protocol papers for stage 1, 2b and 3 studies, and trial registrations for stage 2a studies, were also identified. The number of centres publishing reports and the number of publications has increased each year. Overall, there has been progression through the IDEAL stages; however, about 60 per cent of clinical reports published in 2018 were stage 1 Idea‐type studies.

**Conclusion:**

Since its introduction, studies investigating PIPAC have progressed in line with the IDEAL framework. However, the majority of studies reported recently were stage 0 and 1 studies.

## Graphical abstract

This review assesses whether the introduction of pressurized intraperitoneal aerosol chemotherapy has followed the IDEAL (Innovation, Development, Evaluation, Assessment, Long‐term use) framework, and whether the problems with surgical innovation have been addressed.

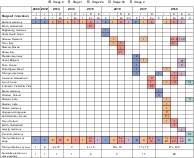

Pipac and review

## Abstract (es)

**Antecedentes:**

El marco conceptual IDEAL (Idea, Desarrollo, Exploración, Evaluación y Estudio a largo plazo) es un esquema de investigación para intervenciones quirúrgicas innovadoras. La quimioterapia intraperitoneal presurizada con aerosol (*Pressurised Intraperitoneal Aerosol Chemotherapy*, PIPAC) es un procedimiento introducido en 2011 y basado en la laparoscopia para administrar quimioterapia intraperitoneal en las metástasis peritoneales. El objetivo de este manuscrito era revisar la literatura sobre PIPAC y evaluar si el desarrollo de la técnica se ha hecho siguiendo el marco IDEAL.

**Métodos:**

Se realizó una búsqueda en Medline y Embase para identificar publicaciones científicas sobre PIPAC aparecidas entre enero de 2000 y febrero de 2019. Los estudios se clasificaron según las etapas IDEAL.

**Resultados:**

Se identificaron 86 trabajos de investigación originales sobre PIPAC. Hubo 23 estudios de la etapa 0, 18 de la etapa 1, 25 de la etapa 2a y 6 de la etapa 2b. También se identificaron protocolos para estudios de las etapas 1, 2b y 3, así como registros de ensayos para estudios de la etapa 2a. El número de centros que publican trabajos y el número de publicaciones ha aumentado cada año. En general, ha habido una progresión a través de las etapas IDEAL; sin embargo, aproximadamente el 60% de los informes clínicos publicados en 2018 fueron estudios tipo “Idea” de etapa 1.

**Conclusión:**

Desde su introducción, los estudios que investigan PIPAC han progresado en la línea del marco IDEAL. Sin embargo, la mayoría de los estudios publicados recientemente fueron estudios de las etapas 0 y 1.

## Introduction

In 2008, the Society of University Surgeons defined an innovative procedure as one that ‘differs from currently accepted local practice, the outcomes of which have not been described, and which may entail risk to the patient’^1^. The IDEAL (Idea, Development, Evaluation, Assessment, Long‐term study) framework is a scheme of investigation for innovative surgical therapeutic interventions proposed by the Balliol Collaboration in 2009^2^, ^3^, ^4^, ^5^ as a strategy to address concerns regarding shortcomings of research in surgery, with particular reference to novel procedures and practices (*Table* 1). The framework requires that surgical innovation should be carried out in a coordinated manner, with investigations progressing to a series of randomized trials. The results of the process should be audited using a clinical registry.

**Table 1:** Summary of the stages of surgical innovation according to the IDEAL paradigm (adapted from references 2 and 5), with description of the interpretation of these stages in this review relating to published work on pressurized intraperitoneal aerosol chemotherapy

Stage of innovation	Description	No. of patients	Proposed method of investigation	Studies in this stage included in this review
0 – Idea	Preclinical work *in vitro* and in animals	None	Varied	Preclinical studies in animals (*in vivo* and post‐mortem models) and *in vitro*
1 – Idea	First human applications: proof of concept and small safety studies	Very few	Structured case reports	Case reports and small case series. Occupational health and safety studies. Scientific studies of clinical samples Data presented relate to safety and/or initial feasibility/proof of concept Prospective or retrospective data collection
2a – Development	Major technical details defined but technique remains experimental	Few, selected	Prospective development studies	Larger case series, and single‐arm non‐randomized studies. Scientific studies of clinical samples Prospective or retrospective data collection
2b – Exploration	Individual learning curves progressing quickly, with resulting increase in patient accrual and broadening of indication. Effectiveness still not demonstrated formally	Many, mixed	Research database, explanatory or feasibility RCT	Large case series from a prospectively maintained database, and RCTs. Scientific studies of clinical samples. Prospective study relating to a new indication for the technique Primary outcomes are efficacy‐related. Prospective data collection
3 – Assessment	Procedure is part of many surgeons' practice and becoming the standard of care	Many, variable	RCT	RCT with primary outcome relating to efficacy
4 – Long‐term	Procedure is routine practice and long‐term outcomes and late/rare complications can be monitored	Almost all	Registry, rare case reports	Not applicable

Pressurized intraperitoneal aerosol chemotherapy (PIPAC) for peritoneal metastases is a laparoscopic procedure used to assess the burden of the peritoneal disease, take biopsies, and deliver intraperitoneal chemotherapy as an aerosol, without increasing the intra‐abdominal pressure^6^. Intraperitoneal administration of chemotherapy for peritoneal metastases from ovarian cancer was developed in the 1970s^7^ and has subsequently been used for many different cancer types in the context of heated intraperitoneal chemotherapy (HIPEC) and early postoperative intraperitoneal chemotherapy (EPIC)^8^. Experimental work^9^, ^10^, ^11^ suggests that both increased temperature and increased pressure can enhance the effect of chemotherapy. The concept of PIPAC was first described in 2000^12^, and was first performed in a patient in 2011^13^.

The aim of this literature review was to assess whether the development and introduction of PIPAC has followed the IDEAL framework.

## Methods

A literature search of the Ovid database was carried out using the terms ‘PIPAC’, ‘ePIPAC’, ‘aerosol$ adj3 chemotherapy’ and ‘pressuri$ adj3 chemotherapy’ on 28 February 2019. The first report describing the concept of a ‘therapeutic capnoperitoneum’ was published in 2000^12^, so the search was limited from 1 January 2000 to February 2019. In addition, the reference lists of identified papers were screened, and http://researchgate.net was searched for the term PIPAC to identify other publications.

Conference abstracts, review articles, articles associated with videos, and book chapters were excluded, as were errata to articles. Only articles in English were reviewed. The full text was then obtained, and the studies were graded according to the stages of innovation set out in the IDEAL paradigm. To obtain an up‐to‐date picture of research activity, http://clinicaltrials.gov and the EU Clinical Trials Register (EudraCT) were searched to identify trials. The results were cross‐referenced with the identified publications. Trials that had not yet been reported in the literature were included and assigned a stage as published protocols. Criteria used to assign the stages are described in *Table* 1.

## Results

### PIPAC: a review of the literature in stages

**Figure 1:**
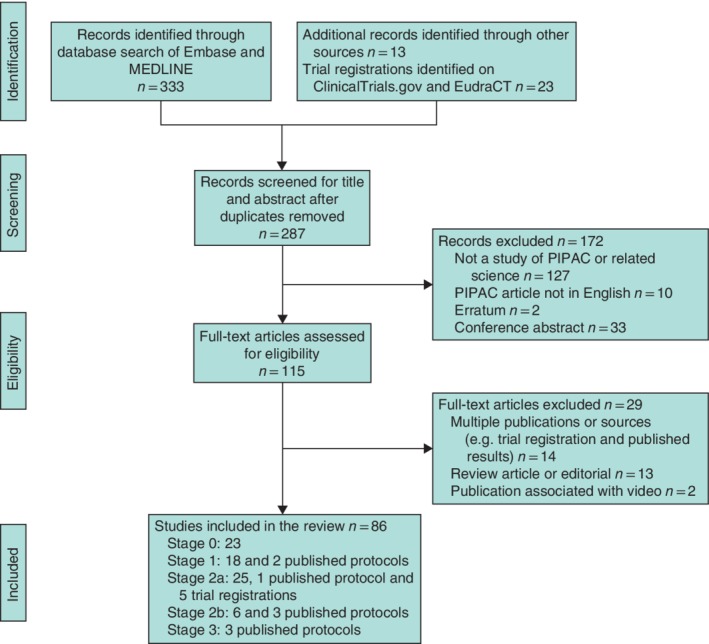
Flow diagram of the literature search and selection of articles for review Search of the EU Clinical Trials Register (EudraCT) and the US National Library of Medicine Trials Register (http://clinicaltrials.gov) is also included. PIPAC, pressurized intraperitoneal aerosol chemotherapy.

The search strategy identified 287 articles after duplicates were removed (*Fig*. 1). Some 172 articles were excluded after review of the title and abstract, and a further 29 were excluded after reviewing the full text. This left 81 original research papers on PIPAC and/or related technology, and five trial registrations.

**Figure 2:**
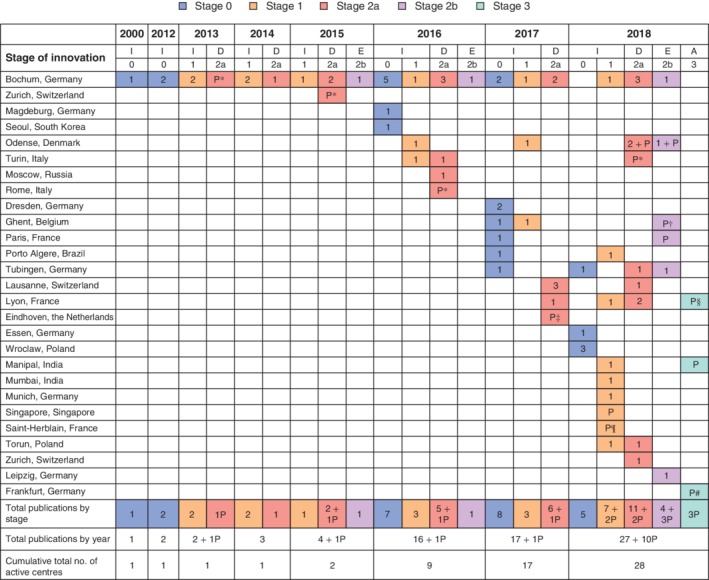
Adoption of pressurized intraperitoneal aerosol chemotherapy according to the IDEAL criteria Studies were identified using the search strategy described in *Fig*. 1. Included studies were then reviewed by a single author and assigned a stage of innovation according to the description of the stage and proposed method of investigation suggested by the IDEAL paradigm, as described in *Table* 1. Study centres are described by the city where the institution of the lead author was located. The number of studies published by each centre is shown, broken down by year and stage of innovation: I, Idea; D, Development; E, Exploration; A, Assessment; P, protocol paper. *Protocol from http://clinicaltrials.gov. †Protocol for multicentre study; other authors are from centres in Lausanne (Switzerland), Odense (Denmark), Paris (France), Tübingen (Germany) and Turin (Italy). ‡Multicentre study; other authors are from centres in Clermont Ferrand and Montpellier (France). §Protocol for multicentre study; other authors are from centres in Berlin, Bochum, Tübingen and Wiesbaden (Germany) and Rome (Italy). ¶Protocol for multicentre study; other authors are from centres in Lyon and Paris (France). #Protocol for multicentre study; other authors are from centres in Berlin, Bochum, Leipzig, Regensburg and Tübingen (Germany) and Geneva (Switzerland).

Overall, the search identified: 23 stage 0 studies^6^, ^12^, ^14^, ^15^, ^16^, ^17^, ^18^, ^19^, ^20^, ^21^, ^22^, ^23^, ^24^, ^25^, ^26^, ^27^, ^28^, ^29^, ^30^, ^31^, ^32^, ^33^, ^34^; 18 stage 1 studies^13^, ^35^, ^36^, ^37^, ^38^, ^39^, ^40^, ^41^, ^42^, ^43^, ^44^, ^45^, ^46^, ^47^, ^48^, ^49^, ^50^, ^51^ and two protocol papers^52^, ^53^ for stage 1 studies; 25 stage 2a studies^54^, ^55^, ^56^, ^57^, ^58^, ^59^, ^60^, ^61^, ^62^, ^63^, ^64^, ^65^, ^66^, ^67^, ^68^, ^69^, ^70^, ^71^, ^72^, ^73^, ^74^, ^75^, ^76^, ^77^, ^78^, one protocol paper^79^ and five trial registrations (NCT01854255, NCT02735928, NCT03246321, NCT02604784 and NCT03124394) for stage 2a studies; six stage 2b studies^80^, ^81^, ^82^, ^83^, ^84^, ^85^ and three protocol papers^86^, ^87^, ^88^ for stage 2b studies; and three protocol papers^89^, ^90^, ^91^ for stage 3 studies. *Fig*. 2 shows the evolution of the literature and the geographical location of authors publishing in this field.

The number of publications increased each year, from five or fewer before 2016, increasing to 17 publications in 2016, 18 in 2017, and 37 in 2018. The cumulative number of active centres has also increased. In general, there has been progression through the stages of innovation, with increasing numbers of Development and Evaluation studies as time passed.

The proportion of papers classified as either IDEAL stage 2a, 2b or 3 has increased over time, with 23 of 37 (62 per cent) of reports published in 2018 describing these types of studies. However, the majority of new centres began their programme of clinical research with a stage 1 Idea study.

The first publication from 11 of 17 centres (65 per cent) that reported clinical results described a stage 1 study, including six of nine centres (67 per cent) that published their first clinical study in 2018.

### Stage 0 – Idea: preclinical work

The potential to deliver drugs by aerosolization into the pneumoperitoneum at laparoscopy was demonstrated initially *in vivo* in a pig model in 2000^12^. Two hypotheses were proposed^12^ to support the technique. The first hypothesis was that intraperitoneal chemotherapy was superior to intravenous chemotherapy for the treatment of peritoneal metastases. The second was that delivery of intraperitoneal chemotherapy as an aerosolized solution to the pressurized pneumoperitoneum would confer pharmacological advantages over chemotherapy in the liquid phase, specifically better distribution around the abdominal cavity and improved penetration into the tissues.

The first studies^6^, ^12^ investigating the technique used methylene blue dye, allowing visual assessment of the distribution and penetration of the aerosol *versus* lavage in an *in vivo* pig model. Distribution of the dye was observed to be superior for aerosolization, although this was assessed by visual inspection. The second stage of preclinical testing involved an *ex vivo* tissue model to assess the penetration of a therapeutic substance, Dbait, into peritoneal tissue from a patient with metastatic endometrial cancer^14^. Dbait penetration was assessed using immunohistochemistry. Nodules treated with a pressurized aerosol had a more homogeneous drug uptake and deeper penetration than nodules treated by lavage.

Following these preclinical experiments, the same team progressed to human applications. Subsequent preclinical studies by other groups suggested that the first generation of PIPAC technology had limitations. Experiments using chemotherapeutic agents, where drug uptake can be measured objectively, found that drug distribution and penetration in *ex vivo* 18, 20 and post‐mortem animal^21^ models was heterogeneous. Although drug was detected in tissue that had not been exposed directly to the aerosol jet, the greatest deposition of the aerosol was opposite to the nebulizer.

Analysis of the aerosol has shown that the droplet size is heterogeneous^23^. One group^24^ has developed and brought to market a second device to enable PIPAC, but there is currently little information on the technical performance of this device, nor on its use outside Brazil.

### Stage 1 – Idea: first human applications

The first human applications of PIPAC were carried out between 2011 and 2013, and the first reports were published in 2013^35^ and 2014^13^. PIPAC was delivered as an off‐label therapy to patients for whom ‘no satisfactory alternative therapy was available’ as a result of progression on systemic treatment or intolerance to systemic treatment. Patients were treated with doxorubicin 1·5 mg/m^2^ in 50 ml 0·9 per cent saline, and cisplatin 7·5 mg/m^2^ in 150 ml 0·9 per cent saline, the doses being set arbitrarily as 10 per cent of the HIPEC doses used at that institution. A formal dose escalation study was not done until 2018^48^. The drugs were administered using the nebulizer device (MicroPump™, Reger Medizintechnik, Rottweil, Germany, until 2015; then CapnoPen®, Capnomed, Villingendorf, Germany, from 2015), and the pneumoperitoneum was then left in a steady state for 30 min. Regressive histological changes were observed in repeat biopsies from consecutive procedures, suggesting efficacy. Mild and moderate adverse events were reported, with patients experiencing fatigue, fever, pain and vomiting after surgery^80^. Pharmacokinetic data were collected; these determined that systemic absorption of the chemotherapy agents was low, although only doxorubicin was monitored^13^.

Data on the occupational health and safety aspects of the technique were collected, with no evidence of platinum contamination in the operating theatres^36^, ^80^. As centres across Europe started performing the procedure, verification of its occupational health and safety was repeated^41^, ^43^, ^44^, ^45^. Similarly, as shown in *Fig*. 2, small case series describing the initial experiences of new centres have been published^40^, ^46^, ^47^, ^49^, ^51^.

### Stage 2a – Development: larger case series

The PIPAC programme at the original centre in Ruhr University Bochum, Germany, continued and further case series were published, including patients with colorectal cancer^78^, ^81^, primary peritoneal cancer^81^, gastric cancer^58^, ^81^ and malignant mesothelioma^81^. In gastrointestinal cancer^78^, oxaliplatin was used at a dose of 92 mg/m^2^. Again, this was an arbitrarily derived dose, and no formal dose‐finding study has been published. A larger series of patients with ovarian cancer included one patient who sustained a life‐threatening bowel perforation; however, this occurred when PIPAC was combined with cytoreductive surgery^74^.

A PIPAC training programme was developed, and sales of the device required to deliver PIPAC were limited to clinicians who had been certified. This programme is now overseen by the International Society for the Study of Pleura and Peritoneum^92^. In addition, clinicians were asked to agree to submit data from all cases to an international registry, managed independently by the University of Magdeburg (NCT03210298).

### Stage 2b – Exploration: expanding the indications

As experience of PIPAC has increased, there has been evolution in the perioperative management of these patients, and in the indications for surgery. The duration of hospital stay has decreased, and in one centre selected patients undergo PIPAC as a day‐case procedure^75^. Additional technology has been applied to the technique by some users. Electrostatic PIPAC (ePIPAC) involves the application of electrostatic precipitation to the abdominal cavity, with the aim of increasing drug deposition and adsorption. In preclinical testing, its use during PIPAC increased the penetration of drug^17^. The use of PIPAC in other scenarios, for example in combination with systemic chemotherapy, is under investigation^57^, ^86^. Although the majority of procedures are still performed where disease has progressed despite conventional treatment, one centre^87^ has proposed a randomized trial that will use PIPAC as adjuvant therapy after resection of high‐risk colorectal cancer. The potential of PIPAC as a downstaging treatment to enable cytoreductive surgery was noted in some of the larger retrospective case series^82^, ^93^, and will be evaluated further in an upcoming RCT^86^.

### The future: Moving to stage 3

As documented in *Fig*. 2, there has been rapid expansion in the published literature since the first human report of PIPAC less than a decade ago, and an increasing number of centres are participating. PIPAC is now in widespread use worldwide. There are a number of RCTs proposed or ongoing^86^, ^89^, ^90^, ^91^, focusing in particular on survival and aiming to determine whether PIPAC could become part of standard care in the treatment of peritoneal disease.

## Discussion

The introduction of PIPAC has, in principle, followed the pattern of investigation advocated by the IDEAL collaboration. However, this review of the development of PIPAC has highlighted some of the difficulties in research in surgical practice. Despite hundreds of reported cases in the literature, the true efficacy of the procedure is still unclear.

In many instances, PIPAC has been initiated outside of a trial. Although centres have published case series of prospectively collected data, there have been multiple publications of data from overlapping time periods, for example in 2014 and 2015^13^, ^38^, ^55^, ^74^ and 2016^40^, ^73^. Formally registered programmes of research have also been conducted, usually consisting of small studies of safety and feasibility initially, followed by larger cohort studies. However, this pattern has been repeated several times across different centres in different countries. This may have been unnecessary in cases where the same drug doses and procedural steps were used, and may have delayed the progression to larger phase II/III studies.

The difficulty of overcoming the technical learning curve associated with a new procedure has been described as a potential barrier to effective research in surgery in the past^3^, ^4^. In the case of PIPAC, there may also be issues relating to patient selection and perioperative care. Other reasons for the duplication of safety studies may relate to individual regulatory requirements in the different countries, or because studies were conceived before the results of earlier trials had been published.

The IDEAL framework recommends that trial protocols are registered publicly, and advocates the reporting of the results from new procedures on online registers available to all surgeons^2^. This recommendation has not been followed by all PIPAC centres. Although 67 clinical studies were published, only 23 of them were registered on http://clinicaltrials.gov and EudraCT.

There is also the issue of the limited funds available for surgical studies. This can result in the duplication of smaller, less powerful, studies, without assessing the effect of the treatment on longer‐term outcomes.

Ethical concerns have been raised in the past about surgical innovation^94^. Three systematic reviews^95^, ^96^, ^97^ have summarized the likelihood of adverse effects after PIPAC, although the lack of efficacy data resulting from controlled trials makes a risk *versus* benefit discussion challenging. There may also be rare complications not identified during early development of the technique, such as the cases of peritoneal sclerosis after PIPAC with oxaliplatin^65^ and severe hypersensitivity reactions to platinum‐based chemotherapy during PIPAC^69^. The IDEAL Collaboration suggest that clinicians use all available data from previous studies to counsel patients and disclose the possibility of unknown or unanticipated side‐effects and complications^5^.

There may be ethical concerns relating to the involvement of the innovator or manufacturer of a new device in ongoing research. In the case of PIPAC, the sale of the MicroPump™/CapnoPen® device was initially limited to clinicians who had been trained by the developer, and a contribution to an independently managed international registry (NCT03210298) was expected. An annual symposium was organized, and latterly this platform for sharing experience and update training has been formalized with the foundation of the International Society for the Study of Pleura and Peritoneum^92^, contributing to standardization of the technique^98^. This may prove useful for the conduct of multicentre trials in the future.

## Disclosure

The authors declare no conflict of interest.
